# Clinical and radiological outcomes following arthroscopic-assisted management of tibial plateau fractures: a systematic review

**DOI:** 10.1007/s00167-014-3256-2

**Published:** 2014-09-24

**Authors:** Hong-Wei Chen, Guo-Dong Liu, Li-Jun Wu

**Affiliations:** 1 Department of Orthopedics, Wenzhou Medical College-Affiliated Yiwu Central Hospital, Yiwu, 322000 Zhejiang People’s Republic of China; 2 Department 8, Research Institute of Surgery, Daping Hospital, Third Military Medical University, Chongqing, 400042 People’s Republic of China; 3 Department of Orthopedics, Wenzhou Medical College-Affiliated Second Hospital, Wenzhou, 325000 Zhejiang People’s Republic of China; 4 Institute of Digital Medicine, Wenzhou Medical College, Wenzhou, 325000 Zhejiang People’s Republic of China

**Keywords:** Arthroscopic, Clinical, Fracture, Outcome, Radiological, Systematic review, Tibial plateau

## Abstract

**Purpose:**

To carry out a systematic review of the literature on arthroscopic-assisted management (all types) of tibial plateau fractures to gain a more comprehensive understanding of clinical outcomes with this surgical technique, specifically to determine whether this may be a viable technique for the management of tibial plateau fractures.

**Methods:**

MEDLINE, Cochrane, and EMBASE databases were searched until July 2013 using combinations of the search terms: tibial plateau, fractures, and arthroscopically/arthroscopic/arthroscopy/percutaneous/minimally invasive. Inclusion criteria were observational study, patients with tibial plateau fractures, and clinical and radiological outcomes assessed using Rasmussen scoring system. The outcome measures of interest were clinical and radiological Rasmussen scores and the prevalence of secondary osteoarthritis.

**Results:**

A total of 12 studies, 5 prospective and 7 retrospective, involving 353 patients were included in the review. The majority of patients in most studies had Schatzker type I–III fractures. The graft material used varied between studies. The length of the follow-up was typically between 34 and 38 months. Mean clinical Rasmussen scores ranged from 25.5 to 28.4. In each study, the majority (≥80 %) of patients had excellent/good clinical Rasmussen scores. In each study, the majority (≥63 %) of patients had excellent/good radiological Rasmussen scores. The proportion of patients who experienced secondary osteoarthritis was variable, ranging from 0 to 47.6 %.

**Conclusions:**

The results of this systematic review indicate that arthroscopic-assisted management of tibial plateau fractures can be effective. Surgeons should consider using this approach when treating patients with tibial plateau fractures.

**Level of evidence:**

III.

## Introduction

Tibial plateau fractures, which are typically caused by high-energy trauma or osteoporosis in older adults, comprise approximately 1 % of all fractures [2, 25]. These fractures are typically characterized using the Schatzker system [23], in which fractures are classified as type I–VI, where type I is indicated by pure cleavage of the lateral plateau, type II is indicated by lateral splitting with depression, type III is indicated by pure depression of the lateral plateau, type IV is indicated by medial plateau fracture with or without an intercondylar fracture, type V is indicated by bicondylar fracture, and type VI is indicated by unicondylar or bicondylar tibial plateau fracture with an extension separating the metaphysis and diaphysis. Tibial plateau fractures are often complex (estimates suggest that 30–35 % are bicondylar) and commonly occur with associated soft tissue injury [2, 25]. As such, treatment can be very challenging for the surgeon [16]. Unsurprisingly, many different surgical techniques and approaches have been described for the management of tibial plateau fractures; however, there is a lack of clear definitive information in the literature as to which is the most appropriate technique [16].

As with any fracture, the aim of surgery in the management of tibial plateau fractures is to restore the normal anatomy, repair soft tissue injuries, and facilitate the return to normal physiological functioning. The two major surgical techniques currently employed for tibial plateau fractures are open reduction and internal fixation (ORIF) and arthroscopic-assisted reduction and internal fixation (AARIF). Being less invasive, AARIF has a clear advantage over ORIF. Evidence suggests that various complications, including pin tract and deep infection, loss of reduction, and septic arthritis are relatively common with ORIF [9, 11, 14]. Of note, overall morbidity has been reported to be lower with AARIF compared with ORIF due to the decreased invasiveness of the approach [2]. AARIF also allows for direct and precise examination of intra-articular lesions. As such, the use of AARIF has been advocated for the management of all tibial plateau fractures [2, 3].

Over the last 10–15 years, the findings from a number of studies have been published reporting on outcomes following AARIF for the management of tibial plateau fractures. To gain a more comprehensive understanding of clinical outcomes and determine whether this may be a viable technique, a systematic review of the literature on arthroscopic management (all types) of tibial plateau fractures was carried out. Specifically, the review focused on identifying studies reporting clinical and radiological outcomes, as well as the occurrence of postoperative osteoarthritis.

## Materials and methods

The PRISMA guidelines for the reporting of systematic reviews and meta-analyses were followed [17]. As this study was a systematic review that did not involve human subjects, Internal Review Board approval was not required.

MEDLINE, Cochrane, and EMBASE databases were searched until July 2013 using combinations of the search terms: tibial plateau, fractures, and arthroscopically/arthroscopic/arthroscopy/percutaneous/minimally invasive. Reference lists of pertinent studies were hand searched to identify other potentially relevant studies.

The inclusion criteria for selection of studies were as follows: observational study; patients had tibial plateau fractures; outcomes were assessed using clinical and radiological scales according to Rasmussen scoring system [20]; and published in English. The exclusion criteria were as follows: clinical outcomes not provided; cadaver studies; or published in the form of a letter, comment, editorial, or case report.

Data were extracted by two independent reviewers who consulted with a third reviewer to resolve any disagreements. Data extracted from eligible studies included the following: first author name; study design; number, sex distribution, and age of patients; Schatzker classification [23]; graft type; length of follow-up; clinical and radiological Rasmussen scores [20]; and the prevalence of secondary osteoarthritis.

The outcome measures of interest were clinical and radiological Rasmussen scores and the prevalence of secondary osteoarthritis.

The Newcastle–Ottawa Scale was used to assess the quality of the studies included in the systematic review.

## Results

### Study selection

**Fig. 1:**
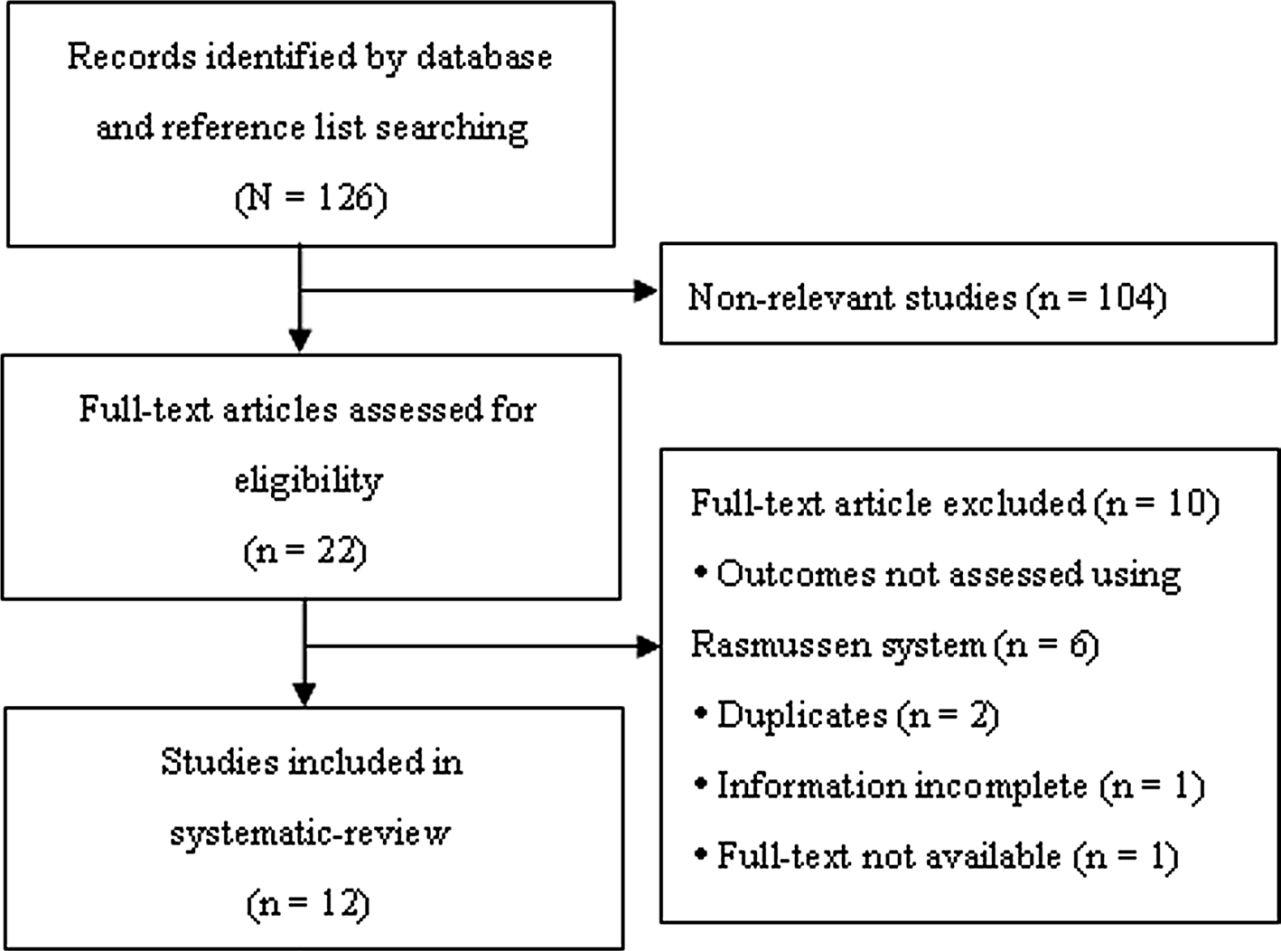
Flow diagram of study selection

Of the 126 articles identified in the search, 104 did not meet the eligibility criteria after abstract review and were excluded; 22 articles underwent full-text review (Fig. 1). Subsequently, 10 articles were excluded and 12 studies were included in the systematic review.

### Study characteristics

The key characteristics of the studies included in the systematic review are summarized in Table 1. Of the studies included, five [4, 5, 8, 15, 22] were prospective studies and seven [1, 6, 12, 19, 21, 24, 26] were retrospective studies. The number of patients included in the studies ranged from 10 to 54 (total = 353). The proportion of male patients in the studies ranged from 10.0 to 75.6 %, with 7 of 12 studies [1, 5, 6, 12, 19, 24, 26] including a majority (>50 %) of male patients. The mean age of patients ranged from 36 to 72 years, although the mean age of patients was in the 41–49 years 7 of 12 studies [4, 8, 12, 15, 22, 24, 26]. The type of Schatzker fracture classification was variable between studies, although the majority of (or all) patients in most studies [4, 6, 8, 12, 19, 21, 22, 24, 26] had type I–III fractures. All patients in the 2003 study reported by Chan et al. [5] had type V or VI fractures, while the majority (>50 %) of patients in the 2008 study reported by Chan et al. [4] had type IV–VI fractures. Alternative fracture classification systems (Association for Osteosynthesis/Association for the Study of Internal Fixation and AO-Müller/Orthopaedic Trauma Association) were used in the studies reported by Lobenhoffer et al. [15] and Asik et al. [1]; the majority (≥89 %) of patients in these two studies had type B1–B3 fractures. The type of graft material used varied between studies. The method of fixation most commonly used (8 of 12 studies) was screw fixation. The length of follow-up was reported in 11 of the 12 studies and ranged from a mean of 24–87 months. A length of follow-up from 34 to 38 months was reported in five studies [1, 6, 12, 21, 26].

**Table 1:** Characteristics of studies included in the systematic review

References	Study design	Patient number	Sex, % male	Mean age (range), years	Schatzker classification	Graft type	Fixation	Mean follow-up (range), months
Rossi et al. [22]	Prospective	46	45.6	48 (25–73)	II (41 %); III (59 %)	Compacted cancellous bone graft	6.5 mm screw	NA
Chan et al. [4]	Prospective	54	46.3	48 (22–868)	I (2 %); II (39 %); III (7 %); IV (19 %); V (15 %); VI (19 %) (closed fracture)	Autogenous iliac crest bone graft or allogeneic bone graft	Kirschner wire	87 (18–128)
Chan et al. [5]	Prospective	18	66.7	35 (23–45)	V (61 %); VI (39 %) (closed fracture)	Autogenous iliac crest bone graft	Kirschner wire	48 (39–69)
Gill et al. [8]	Prospective	25	40.0	45 (17–74)	I (8 %); II (20 %); III (64 %); IV (8 %)	Coral hydroxyapatite bone graft substitute	7.3 mm screw	24 (12–35)
Lobenhoffer et al. [15]	Prospective	10	20.0	49	AO/OTA classification: B1 (20 %); B2 (20 %); B3 (60 %)	Allogeneic bone graft	7.3 mm screw	52 (36–96)
Siegler et al. [24]	Retrospective	27	63.0	45 (18–79)	I–III (closed fracture)	Autograft or bone substitute	6.5 mm screw	59.5 (24–138)
Kayali et al. [12]	Retrospective	21	66.7	41 (23–77)	I–III (closed fracture)	Corticocancellous allograft	6.5 mm screw	38 (12–96)
Duan et al. [6]	Retrospective	39	71.8	36 (17–58)	I (10 %); II (31 %); III (23 %); IV (31 %); V (5 %)	Synthetic, autograft, and allograft	Screw	33.6 (12–60)
Pogliacomi et al. [19]	Retrospective	18	72.2	36 (15–61)	I (22 %); II (33 %); III (33 %); IV (11 %)	Human or synthetic bone grafts	Screw; plate	NA
Asik et al. [1]	Retrospective	45	75.6	39 (15–68)	AO/ASIF classification B1 (20 %); B2 (26); B3 (43 %); C1 (7 %); C2 (2 %); C3 (2 %) (closed fracture)	Autogenous corticocancellous iliac bone graft	Screw	36 (14–72)
van Glabbeek et al. [26]	Retrospective	20	60.0	49 (18–78)	I (35 %); II (50 %); IV (10 %); V (5 %)	Human allograft bone	Screw	39 (27–64)
Roerdink et al. [21]	Retrospective	30	10.0	72 (57–92)	I (20 %); II (43 %); III (17 %); IV (10 %); V (7 %); VI (3 %)	Bone plug or methyl methacrylate	Screw; plate	36 (24–60)

*AO/ASIF* Association for Osteosynthesis/Association for the Study of Internal Fixation, *AO/OTA* AO-Müller/Orthopaedic Trauma Association, *NA* not available

### Clinical outcomes

The clinical outcomes of the studies included in the systematic review are summarized in Table 2. Mean clinical Rasmussen scores were reported in 6 of 12 studies [4–6, 8, 22, 24] included in the systematic review and ranged from 25.5 to 28.4. In the study reported by Roerdink et al. [21], median Rasmussen scores were provided for patients with and without secondary displacement (there was no significant difference in scores between these patients). The distribution of clinical Rasmussen scores (excellent, good, fair, and poor) was reported in 9 of 12 studies [1, 5, 6, 8, 12, 15, 19, 21, 26]. Excellent is indicated by a score of 28–30, good by a score of 24–27, fair by a score of 20–23, and poor by a score <20 [20]. The proportion of patients with excellent, good, fair, and poor scores ranged from 22 to 75, 15 to 67, 4 to 11, and 0 to 10 %. In each study, the majority (≥80 %) of patients had excellent or good scores. Only two studies [4, 24] reported mean radiological Rasmussen scores. In contrast, the distribution (excellent, good, fair, and poor) of radiological Rasmussen scores was reported in 8 of 12 studies [4, 5, 12, 15, 19, 21, 22, 24]. The proportion of patients with excellent, good, fair, and poor scores ranged from 11 to 90, 33 to 96, 4 to 30, and 0 to 11 %. In each study, the majority (≥63 %) of patients had excellent or good scores. More than 30 % of patients in the studies reported by Pogliacomi et al. [19] and Roerdink et al. [21] had fair or poor scores. The proportion of patients who experienced secondary osteoarthritis was reported in 9 of 12 studies [4–6, 12, 15, 19, 21, 22, 24] and ranged from 0 to 47.6 %. The highest proportion of patients experiencing osteoarthritis was reported by Siegler et al. [24], who found that nearly 50 % of patients experienced early osteoarthritis.

**Table 2:** Summary of clinical outcomes for studies included in the systematic review

References	Mean clinical Rasmussen score (range)	Clinical Rasmussen score distribution, %	Mean radiological Rasmussen Score (range)	Radiological Rasmussen score distribution, %	Secondary osteoarthritis, *n* (%)
Rossi et al. [22]	28.2	NA	NA	Excellent (11 %); good (85 %); fair (4 %)	4 (8.6) (tibiofemoral osteoarthritis)
Chan et al. [4]	28.4 (19–30)	NA	16.1 (12–18)	Excellent + good (96 %); fair + poor (4 %)	10 (18.5)
Chan et al. [5]	26.6 (18–29)	Excellent (22 %); good (67 %); fair (11 %)	NA	Excellent (28 %); good (61 %); fair (11 %)	3 (16.7)
Gill et al. [8]	27.5 (21–30)	Excellent (76 %); good (16 %); fair (4 %); poor (4 %)	NA	NA	NA
Lobenhoffer et al. [15]	NA	Excellent (80 %); good (10 %)	NA	Excellent (90 %)	0
Siegler et al. [24]	25.5 (4–30)	NA	8	Excellent (38 %); good (38); fair (19 %); poor (5 %)	10 (47.6) (early osteoarthritis)
Kayali et al. [12]	NA	Excellent (62 %); good (28 %); fair (10 %)	NA	Excellent (52 %); good (33 %); fair (14 %)	5 (24.0)
Duan et al. [6]	26	Excellent (67 %); good (26 %); fair (8 %)	NA	NA	0
Pogliacomi et al. [19]	NA	Excellent (44 %); good (39 %); fair (11 %); poor (6 %)	NA	Excellent (28 %); good (39 %); fair (22 %); poor (11 %)	5 (27.8)
Asik et al. [1]	NA	Excellent (35 %); good (54 %); fair (7 %); poor (4 %)	NA	NA	NA
Glabbeek et al. [26]	NA	Excellent (75 %); good (15 %); fair (5 %); poor (5 %)	NA	NA	NA
Roerdink et al. [21]	Median 9 without secondary displacement 8.7 with secondary displacement	Excellent (40 %); good (40 %); fair (10 %); poor (10 %)	NA	Excellent (20 %); good (43 %); fair (30 %); poor (7 %)	8 (26.7)

*NA* not available

### Quality assessment of studies

Table 3 summarizes the quality assessment of the studies included in the systematic review. The studies were generally found to be of good quality. The majority of studies (10 of 12) included a cohort that was considered to be somewhat representative of the average patient in the cohort. As most (11 of 12) studies were single arm, the second item on the Newcastle–Ottawa Scale was not applicable. Ascertainment of exposure was from a secure record in all 12 studies and all studies demonstrated that the outcome of interest was not present at the start of the study. All studies controlled for outcome scores. With regard to the assessment of outcome, all 12 studies used record linkage. The length of follow-up was considered sufficient in all 12 studies. Likewise, the adequacy of follow-up was considered to be complete in 10 of 12 studies.

**Table 3:** Newcastle–Ottawa Scale quality assessment of studies included in the systematic review

References	Rossi et al. [22]	Chan et al. [4]	Chan et al. [5]	Gill et al. [8]	Lobenhoffer et al. [15]	Siegler et al. [24]	Kayali et al. [12]	Duan et al. [6]	Pogliacomi et al. [19]	Asik et al. [1]	Glabbeek et al. [26]	Roerdink et al. [21]
Selection												
(1) Representativeness of the exposed cohort												
(a) Truly representative of the average patient in the community												
(b) Somewhat representative of the average patient in the community	*	*	*		*	*	*	*	*	*	*	
(c) Selected group of users, e.g., nurses, volunteers				*								*
(d) No description of the derivation of the cohort												
(2) Selection of the non-exposed cohort	NA	NA	NA	NA		NA	NA	NA	NA	NA	NA	NA
(a) Drawn from the same community as the exposed cohort												
(b) Drawn from a different source					*							
(c) No description of the derivation of the non-exposed cohort												
(3) Ascertainment of exposure												
(a) Secure record (e.g., surgical records)	*	*	*	*	*	*	*	*	*	*	*	*
(b) Structured interview												
(c) Written self-report												
(d) No description												
(4) Demonstration that outcome of interest was not present at start of study												
(a) Yes	*	*	*	*	*	*	*	*	*	*	*	*
(b) No												
Comparability												
(1) Comparability of cohorts on the basis of the design or analysis												
(a) Study controls for scores	*	*	*	*	*	*	*	*	*	*	*	*
(b) Study controls for any additional factor												
Outcome												
(1) Assessment of outcome												
(a) Independent blind assessment												
(b) Record linkage	*	*	*	*	*	*	*	*	*	*	*	*
(c) Self-report												
(d) No description												
(2) Was follow-up long enough for outcomes to occur												
(a) Yes (select an adequate follow-up period for outcome of interest)	*	*	*	*	*	*	*	*	*	*	*	*
(b) No												
(3) Adequacy of follow-up of cohorts												
(a) Complete follow-up—all subjects accounted for	*	*	*	*			*	*	*	*	*	*
(b) Subjects lost to follow-up unlikely to introduce bias					*n* = 7*	25%*						
(c) Follow-up rate and no description of those lost												
(d) No statement												

*NA* not applicable

* means yes

## Discussion

This systematic review of the literature on outcomes following AARIF for the management of tibial plateau fracture included a mixture of retrospective and prospective studies, and a total of 353 patients, most of whom had Schatzker type I to III fractures. These findings suggest that, in general, clinical and radiological outcomes are satisfactory following AARIF for tibial plateau fracture management.

Of note, more than 80 % of patients in all studies included in this review had clinical Rasmussen scores that were excellent or good, including one study [5] in which all patients had type V or VI fractures. Radiological outcome findings were less impressive, with patients in only four of the seven studies [4, 5, 12, 15, 22] reporting results having excellent or good Rasmussen scores. The poorer radiological outcomes in the study described by Roerdink et al. [21] may be due to the fact that the patients were far older (mean age = 72 years) than patients in any of the other studies, while the lower scores in the study reported by Siegler et al. [24] may be a reflection of the high rate (48 %) of osteoarthritis among the participants. The reasons for the poorer radiological outcomes in the study reported by Pogliacomi et al. [19] are less obvious, but may be a reflection of surgical technique differences, namely combined arthroscopic and radioscopic-assisted fracture reduction. Taken together, we believe there is a strong evidence that AARIF results in satisfactory (good or excellent) clinical outcomes in a large proportion of patients. Radiological outcomes also appear to be satisfactory in the majority of patients; however, the evidence is not as strong as that for clinical outcomes.

The findings of this review (i.e., generally satisfactory clinical and radiological outcomes as determined by Rasmussen scores) are consistent with the findings from other studies not eligible for inclusion in this review in which different means of assessing clinical and radiological outcomes were used. For instance, Holzach et al. [10] found that 14 of 16 (87.5 %) patients with AO classification-type B2 and B3 fractures had excellent clinical outcomes as determined using the Davos Knee Scoring System and that 12 of 16 (75 %) patients had anatomic alignment on radiography. Using the Hospital for Special Surgery knee score, Hung et al. found that 29 of 31 (93.5 %) patients (generally with Schatzker type II–IV fractures) had satisfactory clinical outcomes and that all fractures exhibited union on radiography. In another study, Kiefer et al. [13] found that 23 of 31 (74.2 %) patients (predominantly with AO type B1–B3 fractures) had excellent or good Lysholm’s knee function scores and that 25 of 31 (80.6 %) patients had anatomic fracture reduction.

All but one of the studies included in this review were single-arm studies; hence, no remarks can be made concerning direct comparison between surgical techniques for the management of tibial plateau fractures, i.e., AARIF vs ORIF. Several studies not eligible for inclusion in this review because they did not report Rasmussen scores, however, have made such comparisons. Specifically, Ohdera et al. [18] found that there were no significant differences in clinical outcomes between patients who underwent AARIF vs those who underwent ORIF; however, postoperative rehabilitation was faster and a higher proportion of patients had anatomic reduction with AARIF compared with ORIF. In another comparative study, Fowble et al. [7] similarly found that the proportion of patients with anatomic reduction was much higher for AARIF (12 of 12; 100 %) compared with ORIF (6 of 11; 55 %). The average length of postoperative hospitalization and time to full-weight bearing were both shorter among patients who underwent AARIF. These patients also experienced fewer and less severe complications [7]. These findings support the notion that AARIF provides more benefits to patients than ORIF for the management of tibial plateau fractures.

Although the primary focus of this review was the assessment of clinical and radiological outcomes, information on the occurrence of postoperative osteoarthritis was also retrieved. This complication was reported in the majority of studies included in the present review, although the prevalence was quite variable. Clearly, this potential complication is one that orthopedic specialists must be acutely aware of. Further research is needed to determine the optimal surgical and postoperative means of minimizing the risk of osteoarthritis with AARIF for tibial plateau fracture repair.

This systematic review has a number of limitations that warrant acknowledgment. Firstly, only 5 of the 12 studies included were prospectively designed; hence, the level of evidence currently available is not particularly high. The potential for retrospective studies to be influenced by various bias-inducing factors cannot be ignored. A second limitation is the fact that all of the studies were quite small scale in terms of patient numbers. This clearly reflects the fact that this type of fracture is not overly common. Finally, there was obvious heterogeneity between studies, in terms of fracture type, the age of patients, the graft material(s) used, fixation technique, and the length of follow-up. All of these factors may have affected the outcomes. Clearly, results from larger-scale, prospective studies would be welcomed.

## Conclusions

The results of this systematic review of the literature support the use of AARIF for the management of tibial plateau fractures. Physicians may therefore consider using this approach when treating patients with tibial plateau fractures.
